# Supplementary material for: Effect of Gossypol on Gene Expression in Swine Granulosa Cells
Source: Toxins (Basel). 2024 Oct 10;16(10):436. doi: 10.3390/toxins16100436 (PMC11511463; doi:10.3390/toxins16100436)
Supplement: Supplementary file 1 [file toxins-16-00436-s001.zip › toxins-3158848-supplementary.pdf]

# Supplementary Materials: Effect of Gossypol on Gene Expression in Swine Granulosa Cells

**Min-Wook Hong, Hun Kim, So-Young Choi, Neelesh Sharma and Sung-Jin Lee**

**Table S1.** Primer sequence for qRT-PCR

| Genes | Porcine | Tm (°C) | Size (bp) | Accession No. |
|---|---|---|---|---|
| ***MMP3*** | F: GATGTTGGTTACTTCAGCAC<br>R: ATCATTATGTCAGCCTCTCC | 59 | 197 | NM_001166308.1 |
| ***CYP1A1*** | F: AATCTTTGAGAAGGGGCGCA<br>R: TCCGACAGCTGGATATTGGC | 60 | 99 | NM_214412.1 |
| ***PRDX6*** | F: GGCCGCATCCGTTTCCACGA<br>R: ACTGGATGGCAAGGTCCCGACT | 60 | 280 | NM_214408.1 |
| ***MGST1*** | F: TGGCAAGGGAGAAAATGCCA<br>R: CATTCAGGTGGGCTCTTCGT | 60 | 77 | NM_214300.2 |
| ***CPEB1*** | F: TTCCAGCACCCTCAGTTAGA<br>R: GAGCTGAAGCCACTGGTATC | 60 | 100 | NM_001097510.1 |
| ***CCNB1*** | F: TTGACTGGCTAGTGCAGGTT<br>R: CTGGAGGGTACATTTCTTCA | 60 | 177 | NM_001170768.1 |
| ***BIRC5*** | F: CCTGGCAGCTCTACCTCAAG<br>R: GAAAGCACAACCGGATGAAT | 60 | 233 | NM_214141 |
| ***COL1A2*** | F: CCTGGCTCTAGAGGTGAACG<br>R: AGCAGGACCAGGATTACCAG | 60 | 246 | NM_001243655.1 |
| ***TGFb3*** | F: CCTGGACCTTTACAACAGCAC<br>R: TTCCTGAGTGCAGTCGTCTC | 60 | 76 | NM_214198.1 |
| ***CDK1*** | F: GGGTCAGCTCGCTACTCAAC<br>R: AAGTTTTTGACGTGGGATGC | 60 | 239 | NM001159304.2 |
| ***SOD3*** | F: CTGTGCTTACCTGCTCCT<br>R: CGAAGTTGCCGAAGTCTC | 56 | 446 | NM_001078688.1 |
| ***β-actin*** | F: GTGGACATCAGGAAGGACCTCTA<br>R: ATGATCTTGATCTTCATGGTGCT | 60 | 131 | U07786.1 |

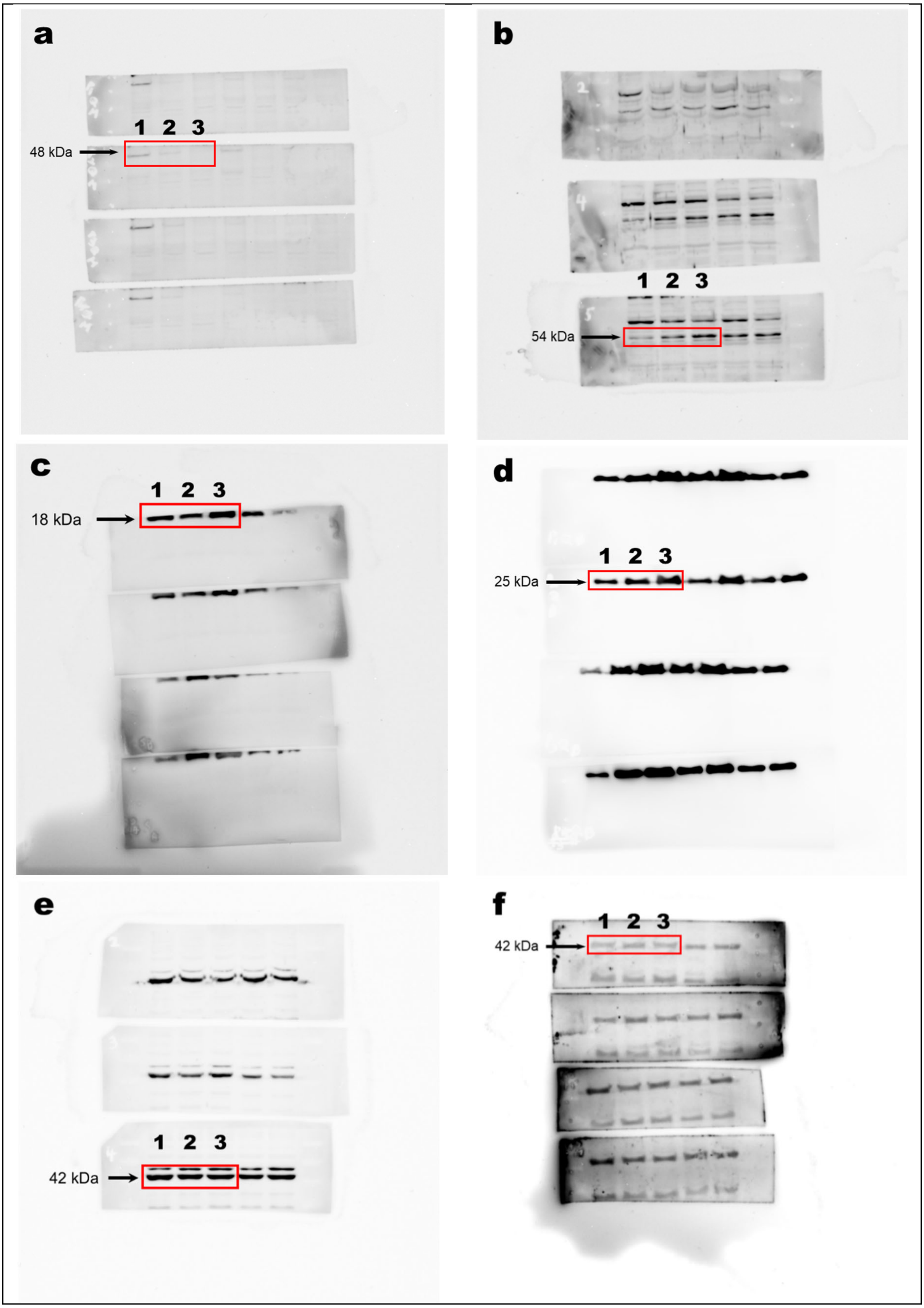


**Figure S1.** Uncropped Western blot images of Figure 6. Red boxed areas show lanes cropped for final figure. (a) CCNB1, (b) MMP3, (c) MGST1, (d) PRDX6, (e) β-actin 1, (f) β-actin 2. 1: GP untreated GCs, 2: GP 6.25 μM treated GCs, 3: GP 12.5 μM treated GCs, GP: gossypol, GCs: granulosa cells
